# Engineering *Saccharomyces cerevisiae* for improved biofilm formation and ethanol production in continuous fermentation

**DOI:** 10.1186/s13068-023-02356-6

**Published:** 2023-07-31

**Authors:** Zhenyu Wang, Weikai Xu, Yixuan Gao, Mingwei Zha, Di Zhang, Xiwei Peng, Huifang Zhang, Cheng Wang, Chenchen Xu, Tingqiu Zhou, Dong Liu, Huanqing Niu, Qingguo Liu, Yong Chen, Chenjie Zhu, Ting Guo, Hanjie Ying

**Affiliations:** 1 State Key Laboratory of Materials-Oriented Chemical Engineering, College of Biotechnology and Pharmaceutical Engineering, Nanjing Tech University, Nanjing, 211816 China; 2 Institute of Industrial Biotechnology, Jiangsu Industrial Technology Research Institute (JITRI), Nanjing, 210032 China; 3 Jiangsu Academy of Agricultural Sciences, Nanjing, 210014 China

**Keywords:** Biofilm, Ethanol, Continuous fermentation, Membrane separation

## Abstract

**Background:**

Biofilm-immobilized continuous fermentation has the potential to enhance cellular environmental tolerance, maintain cell activity and improve production efficiency.

**Results:**

In this study, different biofilm-forming genes (*FLO5*, *FLO8* and *FLO10*) were integrated into the genome of *S. cerevisiae* for overexpression, while *FLO5* and *FLO10* gave the best results. The biofilm formation of the engineered strains 1308-FLO5 and 1308-FLO10 was improved by 31.3% and 58.7% compared to that of the WT strain, respectively. The counts of cells adhering onto the biofilm carrier were increased. Compared to free-cell fermentation, the average ethanol production of 1308, 1308-FLO5 and 1308-FLO10 was increased by 17.4%, 20.8% and 19.1% in the biofilm-immobilized continuous fermentation, respectively. Due to good adhering ability, the fermentation broth turbidity of 1308-FLO5 and 1308-FLO10 was decreased by 22.3% and 59.1% in the biofilm-immobilized fermentation, respectively. Subsequently, for biofilm-immobilized fermentation coupled with membrane separation, the engineered strain significantly reduced the pollution of cells onto the membrane and the membrane separation flux was increased by 36.3%.

**Conclusions:**

In conclusion, enhanced biofilm-forming capability of *S. cerevisiae* could offer multiple benefits in ethanol fermentation.

**Graphical Abstract:**

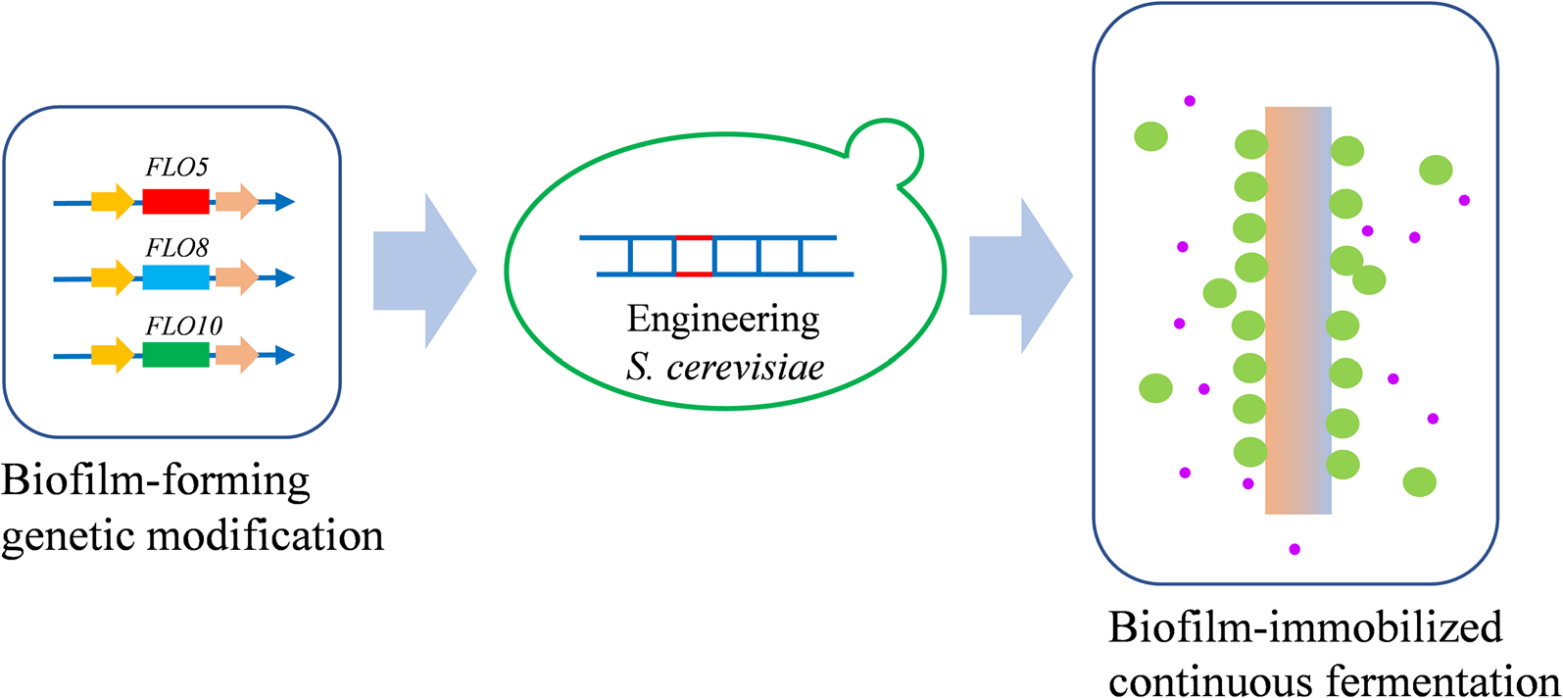

**Supplementary Information:**

The online version contains supplementary material available at 10.1186/s13068-023-02356-6.

## Background

Biofilms are complex multicellular bio-aggregates formed by microbial cells adhered to biotic or abiotic surfaces with the assistance of self-secreted extracellular polymeric substrates (EPS) [1–3]. Biofilms could provide strong protection for cells and improve their tolerance to harsh environments [4]. Although these characteristics of biofilms frequently cause significant health concerns in the medical and food fields, they have many beneficial impacts for industrial processes [5]. Cells in biofilm can maintain growth potential and long-term biological activity, which is widely applied in sewage treatment and immobilized continuous fermentation processes [6–11].

*Saccharomyces cerevisiae* (*S. cerevisiae*) is widely used in industry for ethanol production. It has been reported that *FLO* family genes play a crucial role in *S. cerevisiae* biofilm formation [12]. *FLO* family genes encode glycosylphosphatidylinositol-anchored cell wall proteins that promote cell aggregation. In particular, *FLO1*, *FLO5*, *FLO9* and *FLO10* are considered to facilitate cell–cell adhesion and promote cell flocculation. *FLO11* is considered to facilitate cell-surface adhesion and promote biofilm formation [13]. *FLO8* acts as a transcriptional activator to regulate the expression of *FLO1* and *FLO11* genes [14]. However, the majority of current research has been mainly focused on utilizing plasmids to express *FLO* family genes and investigate their phenotypes, whereas few studies have been conducted to integrate *FLO* family genes into the genome for practical application in industrial production. Although biofilm formation of *S. cerevisiae* could be improved by plasmid-based overexpression of *FLO* family genes, strains harboring plasmids are generally not favorable for continuous industrial production because of plasmid loss. Integrating genes into the genome would avoid this issue and lead to stable and continuous production.

In the continuous fermentation, in situ ethanol separation can eliminate the inhibition effect of high ethanol concentration on cells [15]. Pervaporation membrane separation technology has been widely used for ethanol separation because of its high energy efficiency [16–18]. However, direct contact between the membrane and fermentation broth leads to cells and cellular debris adhered onto the membrane surface, which would eventually pollute membrane and impair separation performance [19, 20]. Biofilm-based immobilization fermentation can greatly reduce the turbidity of fermentation broth and thus the membrane pollution, which provides particular benefits for membrane separation coupled with continuous fermentation [21].

In this study, genome-integrated expression of *FLO5*, *FLO8*, and *FLO10* genes in the industrial strain *S. cerevisiae* 1308 was investigated for their effects on biofilm formation and ethanol production. The engineered strains 1308-FLO5 and 1308-FLO10 effectively improved the biofilm formation, reduced the density of cells dispersed in the fermentation broth and increased ethanol production during the biofilm-immobilized continuous fermentation. When the biofilm-immobilized continuous fermentation coupled with membrane separation, yeast cell contamination of the separation membrane was significantly reduced, and the effectiveness and stability of membrane separation were both improved.

## Results and discussion

### Overexpression of *FLO* genes improved biofilm formation ability

**Fig. 1:**
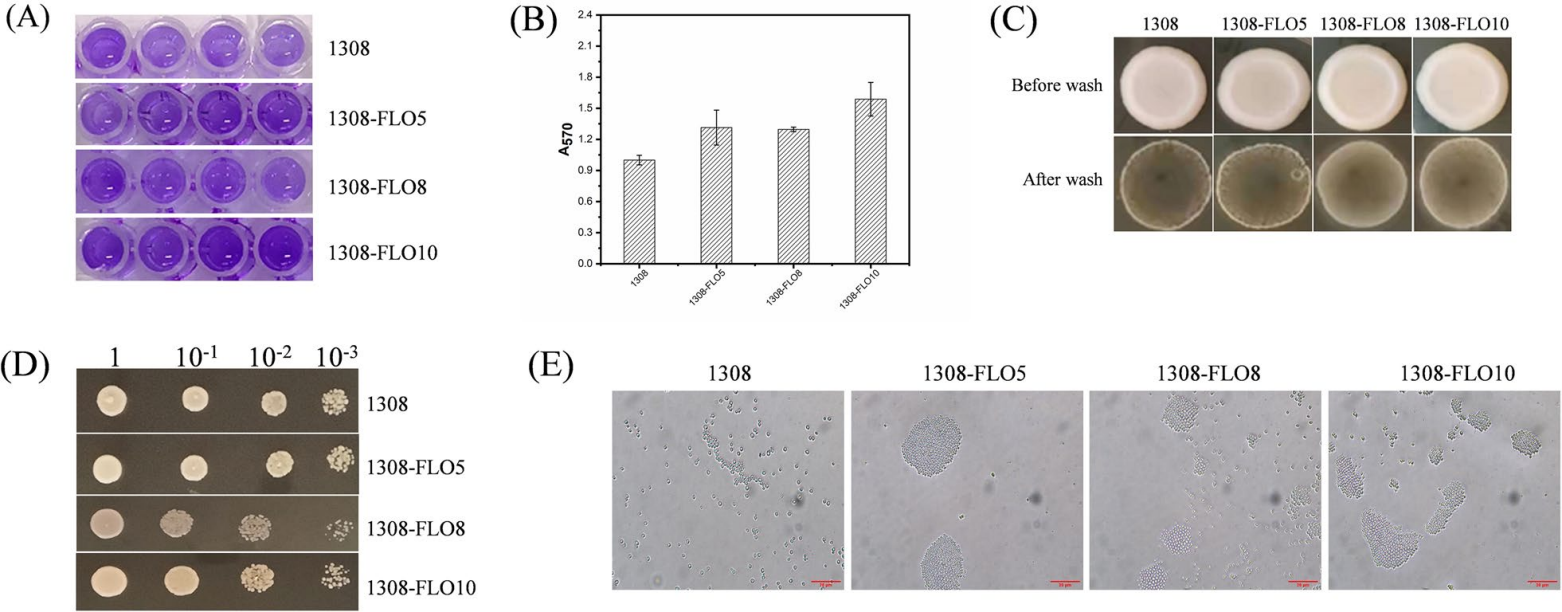
Analysis of biofilm formation capacity. **A** *S. cerevisiae* 1308, 1308-FLO5, 1308-FLO8 and 1308-FLO10 strains were incubated in 96-well plate and biofilms were then stained with crystal violet, darker color means more biofilms. **B** The optical density at 570 nm was measured after the crystalline violet staining of biofilms. **C** Plate invasion capability of *S. cerevisiae* 1308, 1308-FLO5, 1308-FLO8 and 1308-FLO10 strains. **D** Growth of *S. cerevisiae* 1308, 1308-FLO5, 1308-FLO8 and 1308-FLO10 with serially diluted inoculum. **E** Images of aggregation of *S. cerevisiae* 1308, 1308-FLO5, 1308-FLO8 and 1308-FLO10 observed under a microscope, the cells of recombinant strains aggregated, whereas the WT strain was dispersed

To construct a stable biofilm-forming yeast for industrial application, the genes of *FLO5*, *FLO8*, and *FLO10* were integrated into the genome of *S. cerevisiae* under a constitutive promoter *TPI* for overexpression. Recombinant strains were verified and named 1308-FLO5, 1308-FLO8 and 1308-FLO10, respectively. Quantification of biofilm formation by crystalline violet staining revealed that all recombinant strains had a significantly greater capacity for biofilm formation than the wild-type (WT) strain (Fig. 1A). Compared to the WT strain, the biofilm formation of 1308-FLO5, 1308-FLO8 and 1308-FLO10 increased by 31.3%, 29.5% and 58.7%, respectively (Fig. 1B). Additionally, plate invasion experiments and microscopical cell aggregation observation were also carried out to assess the cell adhesion properties [22]. The plate invasion experiments showed that recombinant strains overexpressing *FLO* genes retained more cells on the agar plate after washing, which indicated that the surface adhering ability of the recombinant strains was enhanced (Fig. 1C). Microscopical observation showed that recombinant cells rapidly aggregated and formed clusters, whereas the WT cells were stably dispersed in the liquid, indicating that the cell–cell adhesion of the recombinant strains was improved (Fig. 1E). Thus, overexpression of *FLO* genes effectively promoted cell–cell and cell-surface adhesion, and improved the ability of *S. cerevisiae* to form biofilms.

**Fig. 2:**
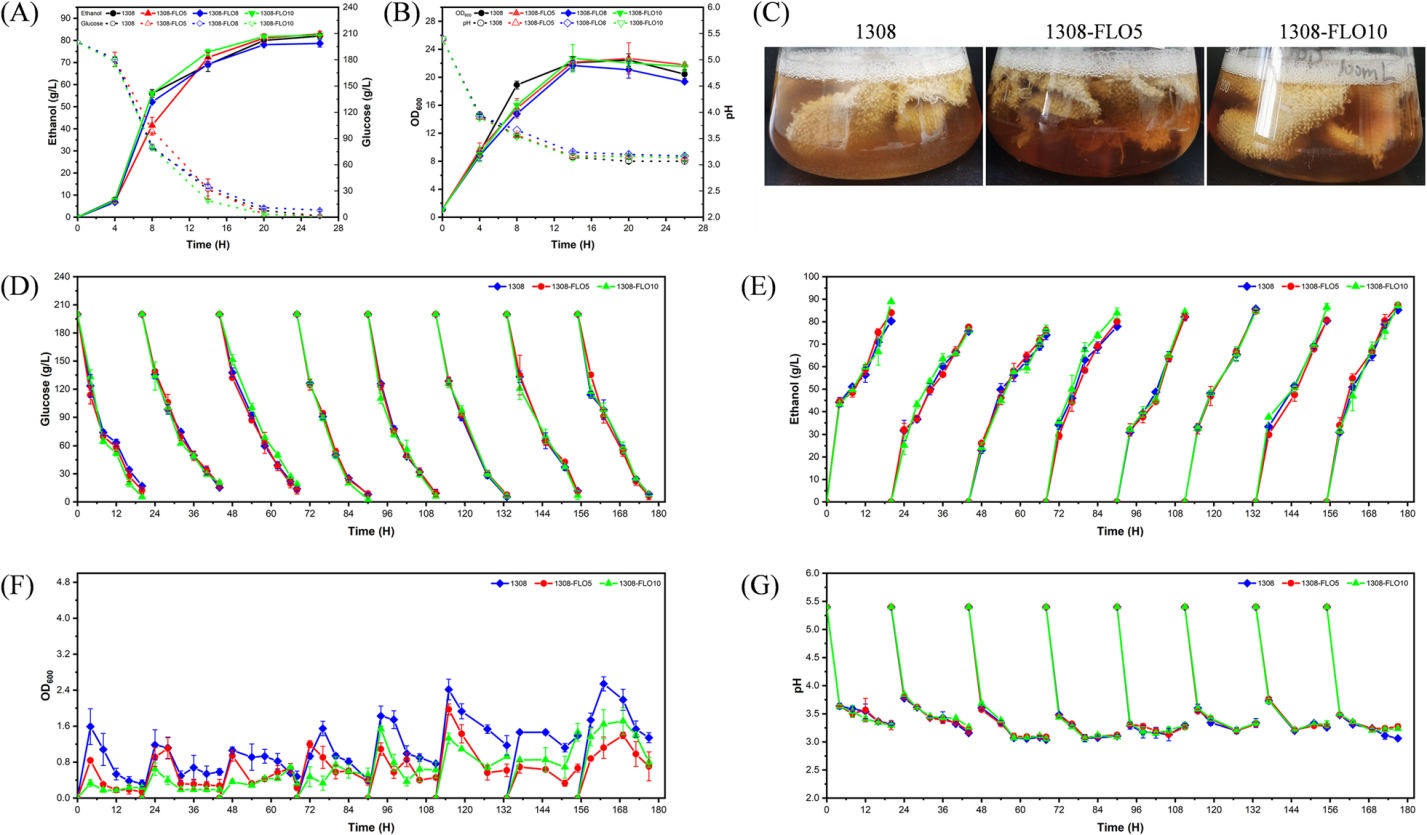
Fermentation kinetics of *S. cerevisiae* 1308, 1308-FLO5, 1308-FLO8 and 1308-FLO10 during free-cell fermentation and biofilm-immobilized continuous fermentation in shake flasks. **A** ethanol concentrations and glucose consumption, (**B**) optical density (OD) at 600 nm, and pH in the free-cell fermentation process. **C** Images of the turbidity of fermentation broth during biofilm-immobilized continuous fermentation in shake flasks by *S. cerevisiae* 1308, 1308-FLO5 and 1308-FLO10. **D** glucose consumption, (**E**) ethanol concentrations, (**F**) optical density (OD) at 600 nm, and (**G**) pH in the biofilm-immobilized continuous fermentation process

Growth ability experiment was also carried out to further confirm whether the strain's growth would be affected by the overexpression of *FLO* genes. There was no significant difference between the growth of 1308-FLO5, 1308-FLO10 and WT strain. However, 1308-FLO8 was weaker than that of WT strain (Fig. 1D). Fermentation experiments also proved that overexpression of the *FLO8* gene caused a reduction in ethanol production (Fig. 2A). In free-cell fermentation, the OD_600_ of 1308-FLO5 and 1308-FLO10 showed no considerable difference from the WT strain, while 1308-FLO8 presented lower OD_600_ than the WT strain (Fig. 2B). This might be due to the fact that *FLO8* is a transcription regulator that regulates multiple genes, elevated levels of these genes possibly enhanced the strain's metabolic load and influenced its growth. Since the 1308-FLO8 strain did not perform as well as the other two recombinant strains did, it was unconsidered in following experiments.

### Production of ethanol by biofilm-immobilized continuous fermentation

To further explore the effect of biofilm enhancement on ethanol production, biofilm-immobilized continuous fermentation was carried out. In shake flasks, compared to free-cell fermentation, biofilm-immobilized continuous fermentation increased the rate of glucose consumption and reduced the fermentation time for all the strains (Fig. 2D). Meanwhile, the ethanol productivity of 1308, 1308-FLO5 and 1308-FLO10 was increased by 15.5%, 17.8% and 20.3%, respectively (Fig. 2E). In the process of biofilm-immobilized continuous fermentation, it was observed that the fermentation broth was becoming increasingly transparent and clear (Fig. 2C). Compared to the WT strain, fermentation broth turbidity of recombinant strains 1308-FLO5 and 1308-FLO10 was decreased by 47.4% and 40.5%, respectively (Fig. 2F), whereas their pH did not differ much (Fig. 2G).

**Fig. 3:**
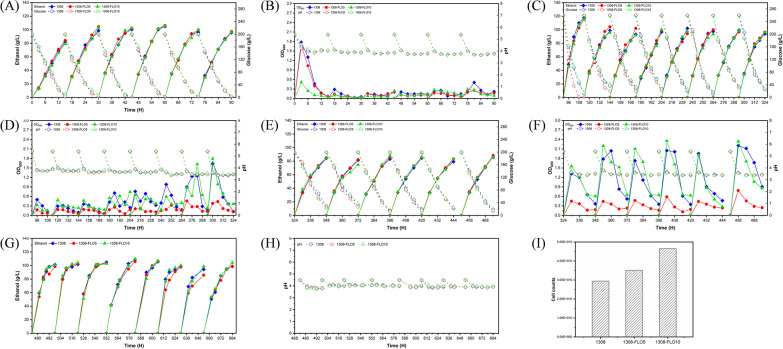
Fermentation kinetics of *S. cerevisiae* 1308, 1308-FLO5 and 1308-FLO10 during biofilm-immobilized continuous fermentation in the 2-L column reactor. **A**, **B** Ethanol production, glucose consumption, optical density (OD) at 600 nm, and pH at 35 ℃ with 200 g/L glucose, (**C**, **D**) ethanol production, glucose consumption, optical density (OD) at 600 nm, and pH at 35 ℃ with 260 g/L glucose, (**E**, **F**) ethanol production, glucose consumption, optical density (OD) at 600 nm, and pH at 37 ℃ with 200 g/L glucose, (**G**, **H**) ethanol production and pH at 35 ℃ and corn mash as a carbon source. **I** The counts of *S. cerevisiae* 1308, 1308-FLO5, and 1308-FLO10 cells that were adsorbed on 1 g of dry cotton fibers after biofilm-immobilized continuous fermentation in the 2-L column reactor

In the 2-L column reactors, the rate of glucose consumption rate was 73.3% higher and the fermentation time was 42.3% shorter in biofilm-immobilized continuous fermentation than in free-cell fermentation (Fig. 3A). The average ethanol production of 1308, 1308-FLO5 and 1308-FLO10 was increased by 17.4%, 20.8% and 19.1% compared with the WT strain in free-cell fermentation, respectively (Fig. 3A). In the biofilm-immobilized fermentation, the fermentation broth turbidity of recombinant strains was decreased by 22.3% (1308-FLO5) and 59.1% (1308-FLO10) compared that of the WT strain (Fig. 3B). At the end of the biofilm-immobilized continuous fermentation, the counts of cells adsorbed on cotton fibers by recombinant strains increased by approximately 19% (1308-FLO5) and 58% (1308-FLO10) compared to the WT strain (Fig. 3I). Results of SEM also showed that more cells of recombinant strains were absorbed on the cotton fiber than the WT strain (Fig. 5A). In addition, the performance of recombinant strains to produce ethanol was further investigated under different fermentation conditions with increased glucose concentration or fermentation temperature. Whether at 35 °C with 260 g/L glucose or at 37 °C with 200 g/L glucose, the average ethanol production of recombinant strains was all higher than that of the WT strain in biofilm-immobilized continuous fermentation (Fig. 3C, E). Additionally, the pH of all strains was almost the same, and the OD_600_ of recombinant strain 1308-FLO10 was lower than those of the WT strain (Fig. 3D, F). These results demonstrated that recombinant strains had robust performance under harsh fermentation conditions.

The fermentation performance of recombinant strains was also investigated in corn mash, which was another industrial raw material and contained many small particles of solids. While the average ethanol production of 1308-FLO5 was comparable to that of WT strain, it was increased by 3.1% in the recombinant strain 1308-FlO10 (Fig. 3G). The main reason for this difference may be that there were a lot of small solid particles in the corn liquid, and the solid residue in the fermentation broth probably had a high shearing force on the biofilm cells. Since the 1308-FLO10 formed more biofilm than 1308-FLO5 as was characterized above, it gave better performance on this raw material. Furthermore, there was no apparent difference of pH between recombinant strain and WT strain during this fermentation (Fig. 3H). Overall, the above results indicated that recombinant strains were able to maintain stable ethanol production and improved production efficiency even in different environments.

### Biofilm-immobilized continuous fermentation coupled with pervaporation membrane separation

**Fig. 4:**
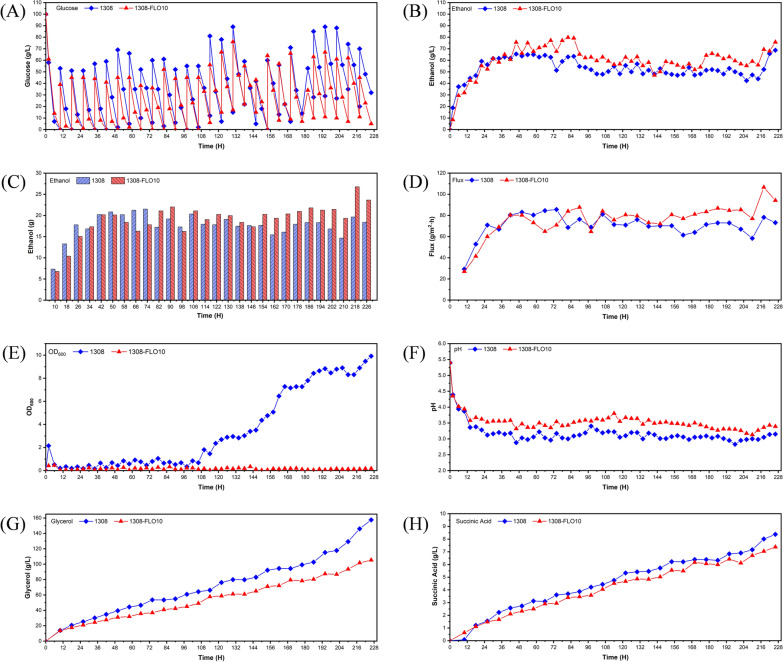
Fermentation kinetics of *S. cerevisiae* 1308 and 1308-FLO10 during biofilm-immobilized continuous fermentation coupled with membrane separation. **A** glucose concentration, (**B**) ethanol production in the 2-L column reactor, (**C**) weight of ethanol after membrane separation, (**D**) membrane separation flux, (**E**) optical density (OD) at 600 nm, (**F**) pH, (**G**) glycerol, and (**H**) succinic acid

**Fig. 5:**
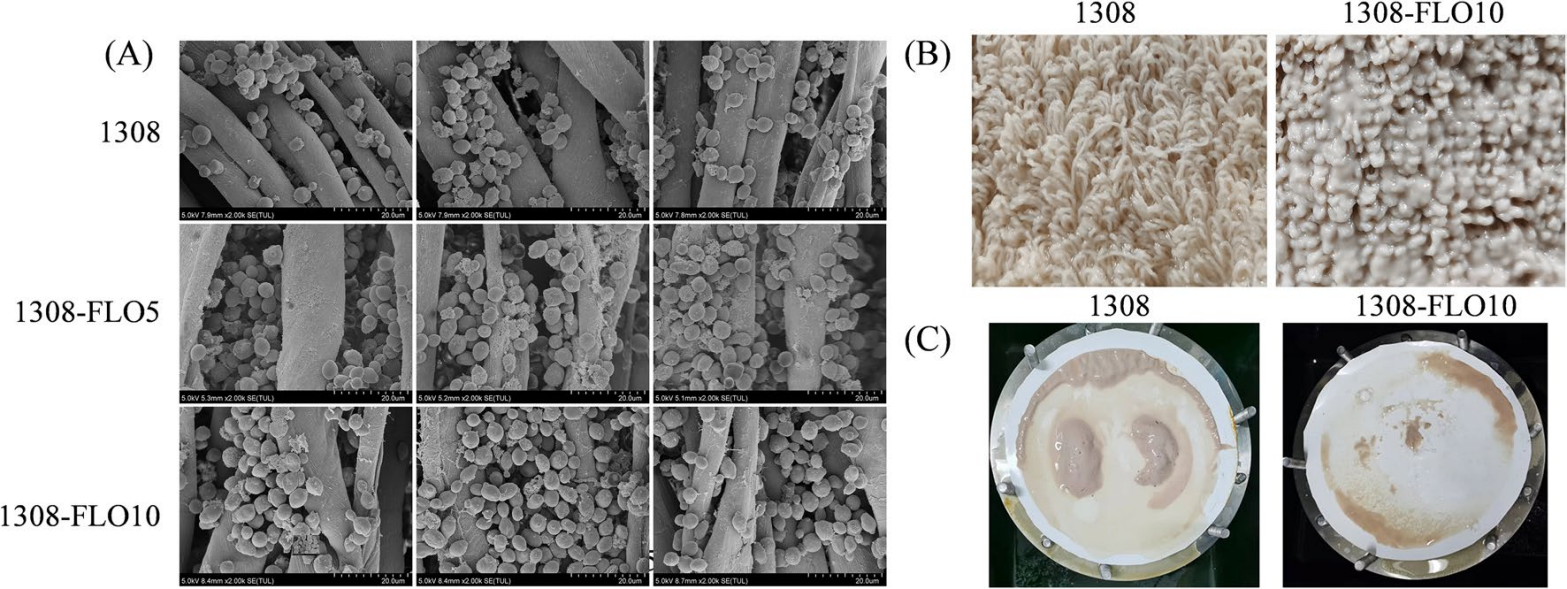
**A** SEM images of biofilms formed on cotton fibers in three different regions after biofilm-immobilized continuous fermentation in a 2-L column reactor. **B** Images of biofilm formed on cotton fibers after biofilm-immobilized continuous fermentation coupled with membrane separation. **C** Images of cells remained on the separation membrane after membrane separation

In biofilm-immobilized continuous fermentation, yeast cells were well immobilized and the turbidity of fermentation broth was greatly decreased, making it suitable for in situ membrane separation. Therefore, biofilm-immobilized continuous fermentation coupled with membrane separation was studied in this research with the recombinant strain 1308-FLO10. During the experiment, the concentration of dispersed free cells in the fermentation broth of the recombinant strain was always lower than that of the WT strain. After 106 h of fermentation, the concentration of free cells in the fermentation broth of WT strain increased sharply and the OD_600_ was steadily increased up to 10. By contrast, the OD_600_ of the fermentation broth for the recombinant strain 1308-FLO10 remained at around 0.2 throughout the fermentation (Fig. 4E). This might be due to the fact that cells shed from the cotton fibers and remained in the fermentation broth. At the same time, the membrane separation flux and ethanol production of the recombinant strain 1308-FLO10 were higher than that of the WT strain (Fig. 4B, D). When the experiment was stopped arbitrarily at 228 h, the production of total ethanol and separated ethanol was increased by 8.3% and 7.3% compared that of the WT strain, respectively (Fig. 4A, C). Also, the ethanol yield of recombinant strain 1308-FLO10 was increased by 5.7% compared that of the WT strain in the fermentation-separation process (Table 1). And the membrane flux was 36.3% higher than that of the WT strain. Strikingly, it was found that only a small quantity of cells of the recombinant strain were attached onto the membrane, whereas a much larger quantity of cells of the WT strain were stacked on the membrane (Fig. 5C). In addition, the biofilm formed by the recombinant strain 1308-FLO10 at the end of fermentation was apparently thicker and much more visible than that of WT strain (Fig. 5B). These results indicated that the recombinant strain 1308-FLO10 cells were well immobilized in the form of biofilm, which reduced yeast cells contamination on the separation membrane and enabled a long-term in situ membrane separation process. It was also found that by-products of the recombinant strain such as glycerol and succinic acid were lower than those of WT strain (Fig. 4G, H). Consistent with this, the pH and ethanol yield of recombinant strain 1308-FLO10 were higher than those of the WT strain (Fig. 4F). These results revealed that the recombinant strain's metabolic flux to ethanol was increased during the membrane separation coupled fermentation.

**Table 1:** Ethanol production and glucose consumption of *S. cerevisiae* 1308 and 1308-FLO10 in biofilm-immobilized continuous fermentation coupled with membrane separation

Strains	Glucose consumed (g)	Separated ethanol (g)	Residual ethanol^a^ (g)	Ethanol in samples^b^ (g)	Total ethanol (g)
1308	1613.38	496.58	68.71	86.28	651.57
1308-FLO10	1654.64	533.04	75.71	96.86	705.61

^a^The weight of ethanol was residue in the 2-L column reactor

^b^The weight of ethanol was residue in samples

## Conclusion

In this study, genome-integrated overexpression of *FLO5, 8, 10* genes in *S. cerevisiae* 1308 effectively increased the ability of biofilm formation, wherein 1308-FlO5 and 1308-FLO10 showed improved production efficiency. The ethanol production of recombinant strain 1308-FLO5 was increased by 20.8% in the biofilm-immobilized continuous fermentation. Cells were well immobilized in biofilm with a low turbidity of the fermentation broth, which significantly reduced cells contamination on the separation membrane and improved separation performance. The membrane flux of the recombinant strain 1308-FLO10 was improved by 36.3% in the biofilm-immobilized continuous fermentation coupled with membrane separation. Engineering *S. cerevisiae* biofilm could be an effective strategy to improve production efficiency.

## Materials and methods

### Strains and growth conditions

*E. coli* DH5α was grown in LB medium with 100 mg/L kanamycin for *pCAS* plasmid construction. An industrial *S. cerevisiae* 1308 was used in this study, which was cultured on yeast extract peptone dextrose (YPD) medium at 30 ℃ [8].

The fermentation medium consists of 200 g/L glucose as a carbon source and other components that were mentioned elsewhere [23]. Seed cultures were grown in a 500-mL shake flask with 100 mL of YPD medium at 30 ℃ and 220 rpm for 20 h. Biofilm-immobilized continuous fermentation of shake flasks was carried out as follows: 10 mL of seed cultures were transferred into a 500-mL shake flask containing 200 mL of fermentation medium with 8 g of dry cotton fibers as an immobilization carrier for cell surface adsorption at 35 °C at a speed of 220 rpm. When the glucose level was below 5 g/L, the old medium was replaced by an equal volume of fresh fermentation medium. Three parallel settings were used for every design and the same protocols for evaluating ethanol, glucose, pH and OD_600_ [24].

Further verification of the recombinant strain's performance in biofilm-immobilized continuous fermentation was necessary. The 2-L column reactor was employed in this research, which contained 70 g dry cotton fibers as immobilization carrier. The 2-L column reactor was sterilized at 121 °C for 20 min. An even mixture of 160 mL of 20-h-old seed culture and 1.6 L of seed medium was pumped into the 2-L column reactor through a peristaltic pump (Longer, BT300-2J, China). Cells were immobilized to the cotton fibers by being circulated through a peristaltic pump at a rate of 30 mL/min, and this process was sustained at 30 ℃ for 14 h. After that, fresh fermentation medium (200 g/L or 260 g/L glucose as a carbon source) was supplemented to the 2-L column reactor in place of the old medium for biofilm-immobilized continuous fermentation at 35 °C or 37 ℃, and the fermentation broth's circulation rate was maintained at the same level as above. The fermentation medium was replaced with a new one when the glucose concentration was less than 5 g/L. To be consistent with industrial production, glucose was replaced by corn mash with 200 U/g of glucoamylase in the 2-L column reactor. Corn mash was obtained by liquified corn as described before [25]. Ethanol, glucose, pH and OD_600_ were detected as described above. After fermentation at 35 ℃ with 200 g/L glucose as a carbon source, a cotton fiber (1 cm^2^) with biofilm cells was obtained from the 2-L column reactor. The cotton fiber was gently washed twice with PBS to remove free cells, and then yeast cells were forcefully eluted from the cotton fiber. The counts of cells were estimated by the value of OD_600_ (one OD_600_ unit was approximately 5 × 10^7^ cells/mL) [26].

### Construction of recombinant yeast strains

*FLO5*, *FLO8* and *FLO10* genes were inserted into 106a or 1622b locus of the *S. cerevisiae* 1308 genome by CRISPR/Cas9 system [27]. This CRISPR/Cas9 system composed of single plasmid (*pCAS*) and donor DNA. The *pCAS* plasmid was constructed as previously described [28], and protospacer adjacent motif (PAM) sequences of *pCAS* plasmid were replaced by the corresponding sequences of 106a (ATACGGTCAGGGTAGCGCCC) or 1622b (GTCACGTTCCTGAGGTTACT) locus [27]. Donor DNA was constructed as follows: the polymerase chain reaction (PCR) was used to amplify terminator (*CYC1*) from the plasmid of *pYES2/CT* and other DNA fragments that contained promotor (*TPI*), upstream homologous sequence, downstream homologous sequence and target genes were obtained from the genome of *S. cerevisiae* 1308, all DNA fragments were ligated together by overlap PCR. Donor DNA and *pCAS* plasmid were transformed into *S. cerevisiae* 1308 according to the previously published electroporation method [27]. The transformed cells were spread onto the freshly prepared YPD solid medium supplemented with 500 mg/L G418 sulfate (Sangon biotech, China). Following incubation at 30 ℃ for 40 h, recombinant strains were screened by colony PCR with the primers 106a-F/106a-R or 1622b-F/1622b-R. All PCR primers used in this study are listed in Additional file 1: Table S1.

### Growth capacity analysis

The single-colony of yeast cells was cultured in 5 mL of liquid YPD medium at 30 ℃ and 220 rpm for 20 h. After diluting 10, 100, and 1000 times with sterile water, one microliter of sample was dripped on YPD solid medium and cultured at 30 °C for 48 h to observe the growth capacity of yeast [29].

### Microtiter plate assay for biofilm quantification

The crystal violet assay was used to evaluate the ability of *S. cerevisiae* to form biofilm [29, 30]. Briefly, yeast cells were harvested and resuspended in YPD medium with an OD_600_ = 1.0 after 20 h of cultivation at 30 °C. A volume of 20 μL of yeast cells and 180 μL of YPD medium were placed into a sterile 96-well plate (Corning, NY, USA), which was cultured for 72 h at 30 ℃, each strain was performed in quadruplicate and the mean value (± SD) was calculated. Following incubation, removed the medium from biofilm-containing wells and gently washed the wells twice with 200 μL of phosphate-buffered saline (PBS) to remove free cells. The biofilms were then stained for 10 min with 200 μL of crystal violet solution (0.1%) at room temperature, repeatedly washed with PBS and allowed to air dry. Finally, 200 μL of acetic acid (33%) was added to every well and incubated at 150 rpm for 30 min at room temperature. The absorbance of 570 nm was measured by a microplate reader (Thermo Scientific™ Multiskan™ FC).

### Plate invasion assays and cell aggregation evaluations

Plate invasion and cell aggregation experiments were performed using yeast cells that had been grown to an OD_600_ of 1.0 as mentioned above. For plate invasion, the yeast cells were dripped on the YPD agar medium and cultured at 30 °C for 72 h. Each plate was then thoroughly rinsed under running water until no colonies were visible, and the pre- and post-wash conditions of the plate were observed to assess the potential for plate invasion.

Considering cell aggregation evaluations, the yeast cells were placed in a 6-well plate with 2.7 mL of fresh YPD media with a sterile cover slip, and the plate was incubated at 30 °C for 72 h. The biofilm cells were fixed with paraformaldehyde (4%) for 30 min at 4 °C after the free cells were removed with PBS. And the cover slips were observed under a microscope (MSHOT, MF52-N).

### Scanning electron microscopy analysis

Following fermentation in the 2-L column reactor, a cotton fiber (1 cm^2^) with biofilms was obtained for scanning electron microscopy (SEM) investigation. The cotton fiber was gently washed twice with PBS and fixed with glutaric dialdehyde (2.5%) and osmic acid (1%) as previously described [31]. After that, cotton fibers were dehydrated with ethanol and dried with the critical point drying method [32]. Finally, treated cotton fibers were photographed by SEM (Hitachi SU 8020, Japan) at 5.0 kV.

### Biofilm-immobilized fermentation coupled with pervaporation membrane separation

**Fig. 6:**
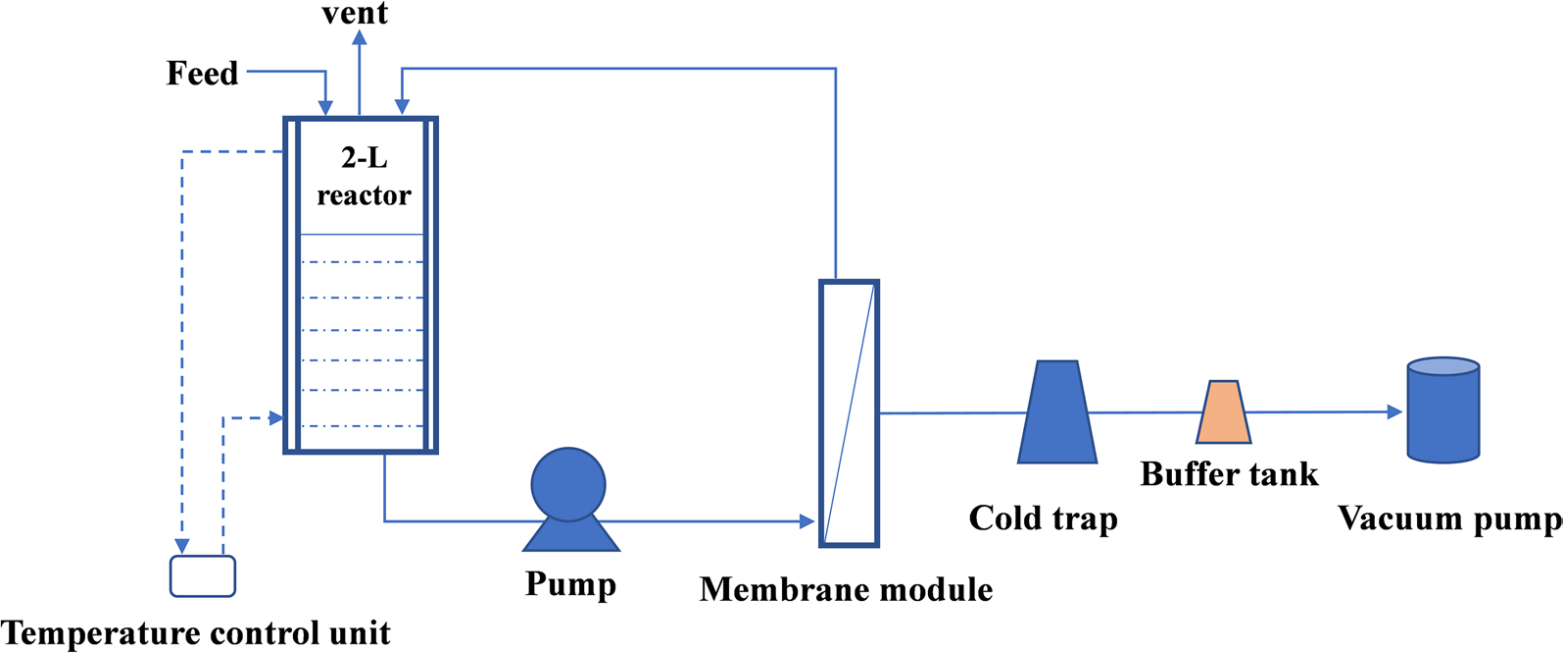
Schematic of the apparatus for the biofilm-immobilized continuous fermentation coupled with membrane separation

The schematic diagram of the biofilm-immobilized continuous fermentation coupled with pervaporation membrane separation system is presented in Fig. 6. The pervaporation membrane was a polydimethylsiloxane (PDMS) membrane, and the module provided an effective membrane area of 314 cm^2^. The PDMS separation membrane was provided by Sichuan University, biofilm-immobilized fermentation was performed as described above. The fermentation broth had a cyclic flow of 450 mL/min on the separation membrane surface. The separation flux *J* was calculated as follows:

$$J=\frac{W}{At},$$

where *J* is the weight of separated ethanol (g). *A* is area of separation membrane (m^2^). *t* is separation time (h).

## Supplementary Information

**Additional** **file** **1:** **Table** **S1**. Sequences of primers used in this study.

## Data Availability

All data generated or analyzed during this study are included in this published article and its Additional files.
