# Supplementary material for: Engineering Saccharomyces cerevisiae for improved biofilm formation and ethanol production in continuous fermentation
Source: Biotechnol Biofuels Bioprod. 2023 Jul 31;16:119. doi: 10.1186/s13068-023-02356-6 (PMC10391976; doi:10.1186/s13068-023-02356-6)
Supplement: Supplementary file 1 — Additional file 1: Table S1. Sequences of primers used in this study. [file 13068_2023_2356_MOESM1_ESM.docx]

**Supplementary material**

**Table S1.** Sequences of the primers used in this study

| Primer name | Primer sequence |
| --- | --- |
| UP-106a-F | TTTGAACCTGTTGCATTGTT |
| UP-106a-R | GAATGACCATACGTAGATCCACAACCGACGATCCGGGG |
| Down-106a-F | CTCGAAGGCTTTAATTTGCAACTAAAAAAATAATAAGG |
| Down-106a-R | ACTTTCTGGCGTTGTCAATG |
| TPI-F(106a) | ACCCCGGATCGTCGGTTGTGGATCTACGTATGGTCATTC |
| TPI-R(FLO5) | CAGTGGTGTGCAATTGTCATTGTTTAGAATTCCTGTATGTGTTTTTTGTAGT |
| TPI-R(FLO8) | CTATTCACTTTATAACTCATTGTTTAGAATTCCTGTATGTGTTTTTTGTAGT |
| FLO5-F | ACTACAAAAAACACATACAGGAATTCTAAACAATGACAATTGCACACCACTG |
| FLO5-R | GGCTTACCTTCGAAGGGCCCTCTAGATTAAATAATTGCCAGCAATAAGG |
| FLO8-F | ACTACAAAAAACACATACAGGAATTCTAAACAATGAGTTATAAAGTGAATAG |
| FLO8-R | GGCTTACCTTCGAAGGGCCCTCTAGATCAGCCTTCCCAATTAATAA |
| CYC1t-F(FLO5) | CCTTATTGCTGGCAATTATTTAATCTAGAGGGCCCTTCGAAGGTAAGCCT |
| CYC1t-F(FLO8) | TTATTAATTGGGAAGGCTGATCTAGAGGGCCCTTCGAAGGTAAGCC |
| CYC1t-R(106a) | CTTATTATTTTTTTAGTTGCAAATTAAAGCCTTCGAGC |
| UP-1622b-F | AACATTTAAGTCACAAGGAG |
| UP-1622b-R | AGAATGACCATACGTAGATCAACTACTTTTCTTAAACTG |
| Down-1622b-F | CGCTCGAAGGCTTTAATTTGCGTAGATACTCGTCTTACGAA |
| Down-1622b-R | ACTTTGGAAAAGAAGGTACG |
| TPI-F(1622b) | ACAGTTTAAGAAAAGTAGTTGATCTACGTATGGTCATTC |
| TPI-R(FLO10) | GGCTTACCTTCGAAGGGCCCTCTAGATTAAACGATTGCCAGTAATAGGG |
| FLO10-F | ACTACAAAAAACACATACAGGAATTCTAAACAATGCCTGTGGCTGCTCGATA |
| FLO10-R | GGCTTACCTTCGAAGGGCCCTCTAGATTAAACGATTGCCAGTAATAGGG |
| CYC1t-F(FLO10) | CCCTATTACTGGCAATCGTTTAATCTAGAGGGCCCTTCGAAGGTAAGCC |
| CYC1t-R(1622b) | TTCGTAAGACGAGTATCTACGCAAATTAAAGCCTTCGAGCG |
| 106a-F | CTATCGTTATCTCCCTGCAT |
| 106a-R | GTACCTGGTAAGTTGACACC |
| 1622b-F | AGTCCATTATCAACAGAAGC |
| 1622b-R. | CACTCTCAAAAACTTGATGT |
